# Mesenteric Paraganglioma: A Case Report and Literature Review

**DOI:** 10.7759/cureus.45685

**Published:** 2023-09-21

**Authors:** Jihoon Lim, Midhir Patel

**Affiliations:** 1 College of Medicine, University of Central Florida College of Medicine, Orlando, USA; 2 Radiology, AdventHealth Orlando, Orlando, USA

**Keywords:** small bowel mesentery mass, paraganglia, mesenteric paraganglioma, mesenteric tumor, extra-adrenal paraganglioma

## Abstract

We report a rare case of a solitary paraganglioma arising from the small bowel mesentery, found in a 70-year-old female who presented with abdominal pain. Paragangliomas are rare neuroendocrine, neural crest-derived tumors, most commonly found in the adrenal medulla. While extra-adrenal paragangliomas arise from diverse locations, mesenteric origins are extremely rare. Our comprehensive review shows 35 previously documented cases and updates the epidemiology, clinical features, and outcomes of mesenteric paragangliomas.

## Introduction

Paragangliomas (PGLs) are rare neuroendocrine neoplasms of neural crest origin that arise from autonomic paraganglia. There are two main types of extra-adrenal paragangliomas: head and neck, and abdominal. The head and neck PGLs are more common and often occur around the carotid artery, jugular vein, and along the base of the skull. Extra-adrenal abdominal PGLs can develop in various locations within the abdomen, including the abdomen's sympathetic and parasympathetic nerve chains.

While a majority of PGLs occur sporadically, approximately 30-40% are associated with hereditary syndromes such as multiple endocrine neoplasia type 2 (MEN2), von Hippel-Lindau disease (VHL), and familial paraganglioma-pheochromocytomas syndrome (HPPS) with mutations identified in several genes (*RET*, *NF1*, *SDHB*, *SDHC*, *SDHD*, and *SDHAF2*).

The incidence of PGLs overall is 1-2/100,000 [1]. However, extra-adrenal PGLs found in the mesentery are exceedingly rare, with one study reporting only 12 documented cases [2]. We report a rare case of extra-adrenal paraganglioma presenting as a mesenteric mass. In addition, we present a comprehensive review of documented cases to update the epidemiology, clinical features, and outcomes of mesenteric paragangliomas.

## Case presentation

A 70-year-old female patient with a past medical history of sarcoidosis and known mesenteric mass presented with increasing abdominal pain, nausea, diarrhea, and emesis. The mass was first noted six years earlier, however, the patient remained asymptomatic and was monitored regularly with imaging. She had no history of colon cancer and her most recent colonoscopy revealed polyps.

**Figure 1:**
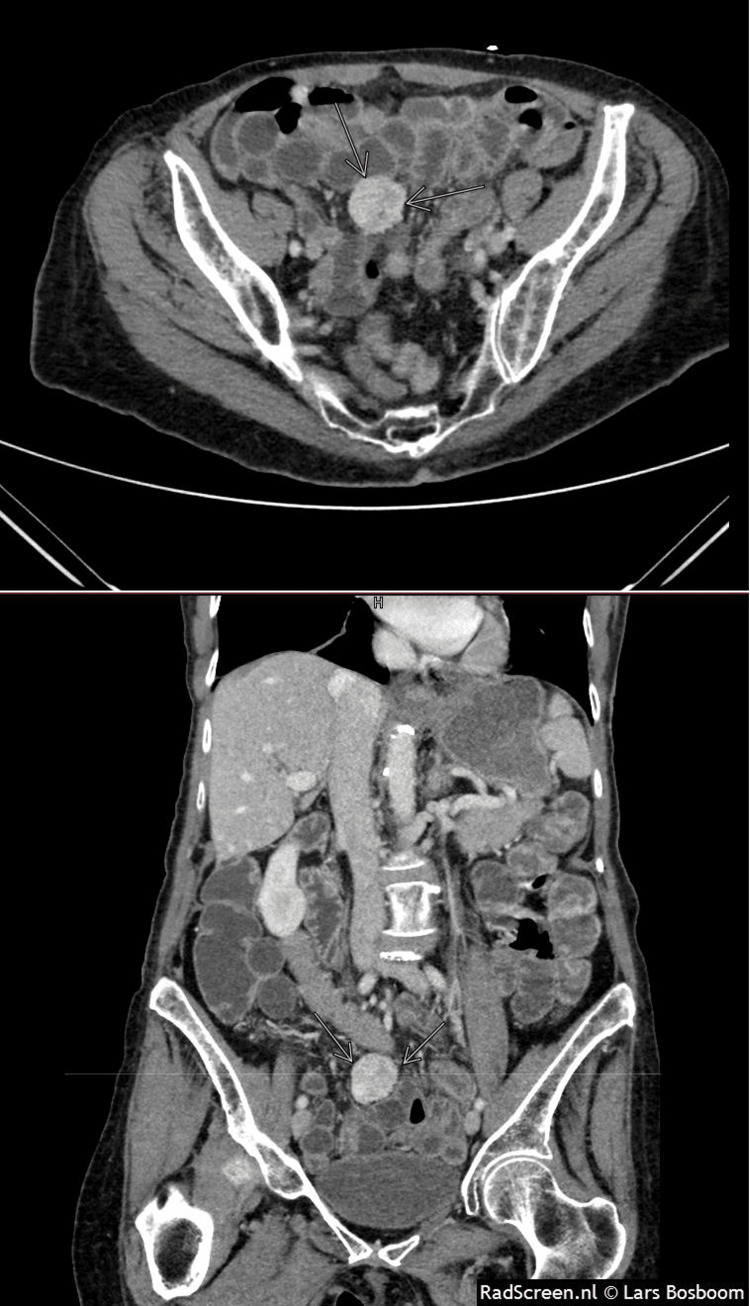
CECT images of the abdomen Axial (top) and coronal (bottom) intravenous (IV) contrast-enhanced computed tomography (CECT) scan images showing an enhancing mesenteric mass.

Computed Tomography (CT) of the abdomen pelvis with intravenous (IV) contrast revealed a right para-midline enhancing mass measuring 2.7 x 2.8 x 3.1 cm in the pelvis. The mass appeared mobile as it was in a different position on prior CTs (Figure 1).

**Figure 2:**
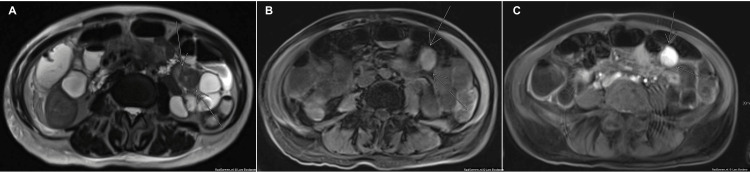
MRI scanning results A) MRI T2 haste axial image showing T2 hypointense mesenteric mass. B) MRI T1 fat-saturated pre-contrast axial image showing T1 isointense mesenteric mass. C) MRI T1 fat-saturated post-contrast axial image showing enhancing mesenteric mass.

Magnetic resonance imaging (MRI) showed a T2 hypointense, T1 isointense mesenteric mass measuring 2.4 x 2.9 x 3.0 cm associated with small bowel loops (Figures 2A-2C).

**Figure 3:**
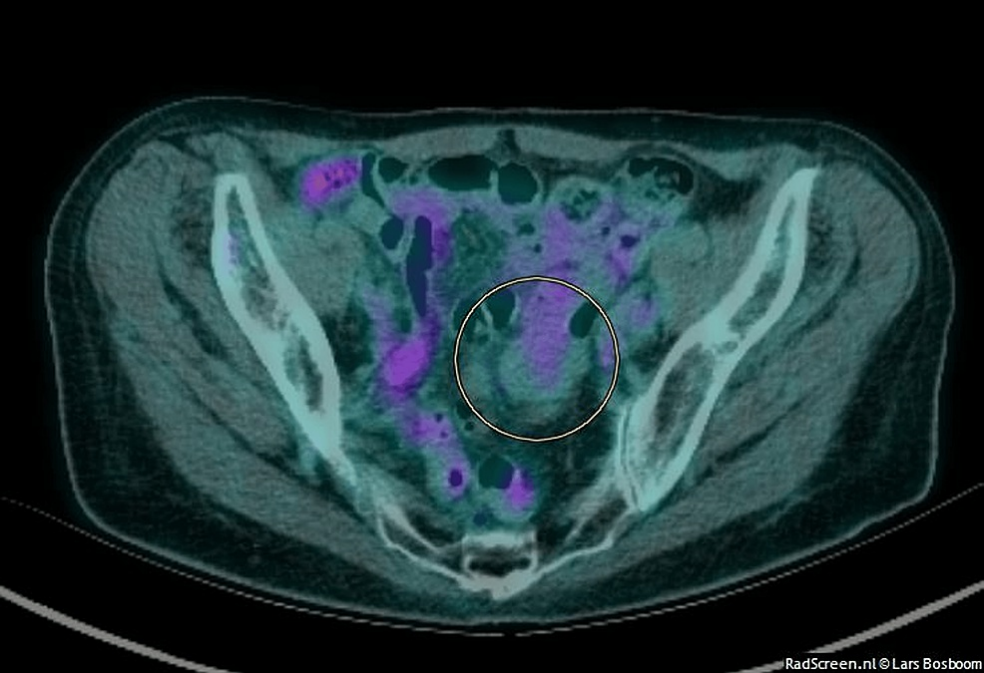
Axial fused 18F-FDG PET/CT image The circle highlights the background level uptake in the solid mesenteric mass. 18F-FDG PET/CT: Positron emission tomography (PET) with 2-deoxy-2-(fluorine-18) fluoro-D-glucose integrated with computed tomography

Additionally, imaging with positron emission tomography (PET) with 2-deoxy-2-[fluorine-18] fluoro-D-glucose integrated with computed tomography (18F-FDG PET/CT) demonstrated low-grade neoplastic activity in the mass (Figure 3).

**Figure 4:**
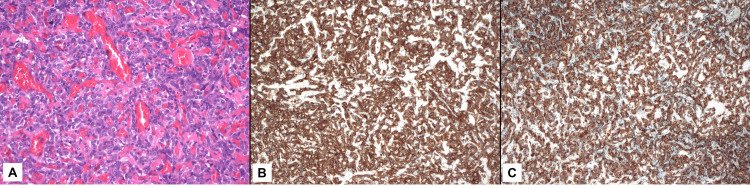
Immunohistochemical study results (A) High-power hematoxylin-eosin preparation at 200X magnification demonstrates a highly vascular tumor consisting of epithelioid cells arranged in a mildly trabecular pattern. Tumor cells are strongly positive for neuroendocrine markers synaptophysin (B) and chromogranin (C), shown at 100x magnification.

Given the documented interval increase in size, the patient was taken to surgery, where a laparoscopic resection of the mesenteric mass and small bowel resection were conducted without complications. The tissue pathology revealed a nodular red-gray mass involving the small intestinal mesentery. Microscopic examination revealed a highly vascular tumor with ovoid tumor cells showing moderately enlarged nuclei with open chromatin. Immunohistochemical stains demonstrated tumor cells to be positive for synaptophysin and chromogranin, indicating neuroendocrine differentiation. Additionally, tumor cells were weakly positive for the *GATA-3* gene and showed rare cells positive for S100. Notably, tumor cells were negative for cytokeratin stains AE1/AE3 and CAM 5.2 (Figures 4A-4C). Therefore, overall findings supported the diagnosis of paraganglioma. Additionally, resection edges and two lymph nodes were negative for the tumor.

## Discussion

We describe a case of small-bowel mesenteric paraganglioma causing abdominal discomfort. We also perform a comprehensive review of the literature using the MEDLINE database as indexed by PubMed with the following search string: “mesenteric” AND “paraganglioma.” A total of 60 articles were screened by title and abstract. Twenty-five articles were subsequently excluded if they were cases of paragangliomas in other regions or of different tumors. A comprehensive list of the 35 included cases of mesenteric paragangliomas is summarized in Table 1.

Our review found 36 documented cases of mesenteric paraganglioma, including our current case. Mesenteric paragangliomas occur most commonly in older adults with an average age of 57 years, with a predominance in females (75%). The most common presenting symptom was a palpable abdominal mass (12/36 cases, 33%), but many patients presented with abdominal pain/discomfort (10/36 cases, 28%) or were asymptomatic (10/36 cases, 28%).

The majority of these tumors are benign, but they can still cause significant health issues depending on their size and location. Sympathetic-associated PGLs can often produce hormones such as catecholamines (e.g., adrenaline and noradrenaline), which can lead to symptoms such as high blood pressure, rapid heart rate, and other signs of excessive sympathetic nervous system activity. Malignant PGLs are less common but are often indistinguishable by imaging. Of the cases that reported metastases in our review, 5/28 (18%) found evidence of PGL in nearby lymph nodes.

While a majority of extra-adrenal PGLs are nonfunctional and discovered incidentally during imaging evaluations for other reasons. CT features include a nonspecific soft-tissue density similar to other neoplasms. Therefore, preoperative diagnosis of extra-adrenal PGLs is usually difficult [3]. MRI characteristics for PGLs are typically low signal intensity on T1-weighted images and strong enhancement after administration of contrast material. While our case showed a hypo-intense lesion, they are typically hyper-intense on T2-weighted images [4].

While only 25% of PGLs are functional and have the potential to secrete catecholamines, 131 I metaiodobenzylguanidine (MIBG) scintigraphy is a specific imaging study for preoperative diagnosis [5]. Furthermore, modern imaging with 68Ga-DOTATATE PET/CT is an accurate technique to diagnose neuroendocrine as it has a reported sensitivity of 83-97% and specificity of 80-100% [6].

The primary treatment choice for PGLs is surgical resection. There have been no reported cases of tumor recurrence, but median follow-up was relatively short. While chemotherapy and radiotherapy may be used for unresectable or metastatic cases, there is no current evidence of increased survival with these treatments [7].

**Table 1:** Epidemiology, clinical features, and outcomes of previously documented cases of mesenteric paraganglioma including the current case

Author	Gender	Age	Associated Symptoms	Pre-operative diagnosis	Tumor size (cm)	Metastasis
Areán et al., 1956 [8]	Male	32	Nausea, vomiting, Diarrhea	Abdominal mass	10	None
Carmichael et al., 1970 [9]	Female	62	Nausea, vomiting, pain	Abdominal mass	3.2	None
Tanaka et al., 1991 [10]	Female	29	Nausea, vomiting	Abdominal mass	10x9x7	Liver
Ishikura et al., 1996 [11]	Female	33	Abdominal pain	Ovarian tumor	15x15x15	Unknown
Onoue et al., 1999 [12]	Female	38	None	Mesenteric tumor	4.5x3.2x3	None
Jaffer and Harpaz, 2002 [13]	Female	76	Abdominal mass, vomiting	Abdominal mass	8.5x8x2	None
Muzaffar et al., 2002 [14]	Female	76	Abdominal mass	Abdominal mass	20x15	None
Ponsky and Gill, 2002 [15]	Female	35	Abdominal mass, headache	Abdominal mass	5.5	Unknown
Canda et al., 2004 [16]	Male	70	None	Mesenteric tumor	18	None
Nobeyama et al., 2004 [17]	Male	53	Abdominal mass	Abdominal mass	15x10x7	Unknown
Kudoh et al., 2005 [18]	Female	72	Abdominal mass, pain	Mesenteric tumor	10x10x9	None
Matsumoto et al., 2006 [19]	Female	77	Abdominal mass	Mesenteric tumor	7x5.5	Unknown
Svajdler et al., 2007 [20]	Male	65	Abdominal mass	Mesenteric tumor	12x9x8	None
Guo et al., 2009 [21]	Female	22	Abdominal mass	Pelvic tumor	11.5x6x11.5	None
Jacob et al., 2012 [22]	Female	63	Lethargy	Abdominal mass	10	Unknown
Chetrit et al., 2012 [23]	Male	55	None	Abdominal mass	11 x 9 x 6	Lymph node
Baker et al., 2012 [24]	Female	64	Abdominal pain	Abdominal mass	4x3	
Fujita et al., 2013 [2]	Female	78	None	Mesenteric tumor	3x1.5x1.5	None
Ozkan et al., 2014 [25]	Female	54	Abdominal mass, pain	Mesenteric tumor	6	None
Pedroso Célia et al., 2015 [26]	Female	32	Abdominal mass	Ovarian mass	11 x 6 x 5	None
Mohd Slim et al., 2015 [27]	Female	69	Abdominal mass, pain	Ovarian mass	18 x 15 x 11	Lymph node
Reyna-Villasmil et al., 2015 [28]	Female	51	Postcoital bleeding	Mesenteric tumor	8 x 6 x 4	None
Kolokotronis et al., 2016 [29]	Female	72	Abdominal pain	Mesenteric tumor	5 x 8	None
Granger et al., 2017 [41]	Female	71	Abdominal discomfort	Abdominal mass	9 x 5 x 10	None
Lee et al., 2017 [30]	Male	70	None	Abdominal mass	15	Lymph node
Ouahabi et al., 2017 [31]	Female	58	Abdominal pain	Abdominal mass	14 x 12	None
Pirpiris et al., 2017 [32]	Female	66	Right-sided flank pain	Mesenteric mass	4.2	None
Ferreira et al., 2018 [33]	Female	32	Abdominal pain	Ovarian mass	6 x 6 x 5	None
Ilieusiu et al., 2020 [34]	Female	64	Bowel Obstruction	Abdominal mass	8 x 6	None
Suzuki et al., 2020 [35]	Male	75	Abdominal pain	Colon Carcinoma	3.5 x 3.5	Lymph node
Rocco et al., 2020 [36]	Female	65	None	Mesenteric mass	3.7	None
Stinson and Bjordahl, 2021 [37]	Female	61	None	Superior mesenteric artery aneurysm	n/a	Unknown
Bardol et al., 2022 [38]	Female	37	Arterial resistant hypertension	Retropancreatic celio-mesenteric mass	6	None
Soler-Silva et al., 2023 [39]	Female	55	None	Abdominal mass	7x8x6	None
Rzepka E, 2023 [40]	Male	63	None	Abdominal mass	13 x 10 x10	None
Current Case, 2023	Female	72	Abdominal pain	Mesenteric mass	3.5 x 3.5 x 2.8	None

## Conclusions

We report an extremely rare case of a solitary paraganglioma arising from the small bowel mesentery, found in a 70-year-old female who presented with abdominal pain. A comprehensive literature review found only 36 reported cases of mesenteric paragangliomas, including the current case. Although rare, these tumors should be among the preoperative differential diagnosis of abdominal masses of unknown etiology.
